# Lattice Discontinuities of 1T-TaS_2_ across First Order Charge Density Wave Phase Transitions

**DOI:** 10.1038/s41598-019-43307-2

**Published:** 2019-05-08

**Authors:** Wen Wang, Dirk Dietzel, André Schirmeisen

**Affiliations:** 1 School of Mechanical Engineering, Southwest Jiaotong University, 610031 Chengdu, China; 2 Institute of Applied Physics, Justus-Liebig-Universität Giessen, 35392 Giessen, Germany

**Keywords:** Phase transitions and critical phenomena, Structural properties

## Abstract

Transition metal dichalcogenides are lamellar materials which can exhibit unique and remarkable electronic behavior due to effects of electron-electron and electron-phonon coupling. Among these materials, 1T-tantalum disulfide (1T-TaS_2_) has spurred considerable interest, due to its multiple first order phase transitions between different charge density wave (CDW) states. In general, the basic effects of charge density wave formation in 1T-TaS_2_ can be attributed to in plane re-orientation of Ta-atoms during the phase transitions. Only in recent years, an increasing number of studies has also emphasized the role of interlayer interaction and stacking order as a crucial aspect to understand the specific electronic behavior of 1T-TaS_2_, especially for technological systems with a finite number of layers. Obviously, continuously monitoring the out of plane expansion of the sample can provide direct inside into the rearrangement of the layer structure during the phase transition. In this letter, we therefore investigate the *c*-axis lattice discontinuities of 1T-TaS_2_ by atomic force microscopy (AFM) method under ultra-high vacuum conditions. We find that the *c*-axis lattice experiences a sudden contraction across the nearly-commensurate CDW (NC-CDW) phase to commensurate CDW (C-CDW) phase transition during cooling, while an expansion is found during the transition from the C-CDW phase to a triclinic CDW phase during heating. Thereby our measurements reveal, how higher order C-CDW phase can favor a more dense stacking. Additionally, our measurements also show subtler effects like e.g. two expansion peaks at the start of the transitions, which can provide further insight into the mechanisms at the onset of CDW phase transitions.

## Introduction

During the past decades, it was found, that first order phase transitions in quasi-two-dimensional (2D) materials can lead to a variety of interesting phenomena, such as superconductivity, occurrence of charge density waves, or friction anomalies^1–6^. Fundamentally, these first order phase transitions are often characterized by a close correlation between the structural and electronic properties driven by interactions based on electron-electron and electron-phonon coupling, which result in periodic distortions of the lattice and modified spatial distribution of the charge density^7^. Among the 2D transition metal dichalcogenides, which are especially relevant in this context, 1T-TaS_2_ is particularly interesting: Depending on temperature, thickness, external pressure and electromagnetic fields, a unique variety of different phases and electronic states can be observed, the latter including a metallic phase, Mott insulation, superconductivity, and four different CDW-states^2,8–11^. This makes 1T-TaS_2_ an important candidate to obtain further fundamental insight into the effects of different electron-electron and electron-phonon coupling mechanism in solid state physics^7,12–14^.

At the same time, charge density wave materials (and in particular 1T-TaS_2_) have attracted a lot of intense studies over the past few years regarding their potential role in designing completely new integrated microelectromechanical systems^11,15–17^. Researchers have e.g. highlighted systems like a room temperature charge density wave oscillator^16^, phase transition transistor^17^, while at the same time, also application in novel concepts of energy-storage is feasible^18^. Also in this context, fully understanding the mechanisms and characteristics of the phase transitions becomes more and more important and urgent.

In principle, any changes of the electronic properties within the CDW-states of 1T-TaS_2_ are predominantly attributed to the reorientation of the Ta-atoms as a consequence of changing electron/phonon coupling. However, in recent years, growing interest was also directed towards understanding the importance of interlayer coupling and stacking order on the electronic properties^19–24^, an effect that can be especially relevant for nanodevices with only few layers of 1T-TaS_2_^16^.

Apparently in this context, accurately measuring the *z*-axis lattice discontinuity across a phase transition, can be considered as a fundamental approach providing not only direct insight into the mechanisms of the first order phase transitions but these out of plane expansion effects should also reflect changes in the stacking order of the material. However, up to now, only very few measurements exist for CDW transitions of 1T-TaS_2_, where results from diffractometry and capacitance dilatometry are available only under ambient pressure. According to their results, the *c*-axis lattice (i.e. the *z*-direction perpendicular to the layers) experiences a sudden expansion during the transition from the nearly commensurate CDW structure to the commensurate CDW structure upon cooling and a sudden contraction during the transition from the commensurate CDW structure to the triclinic CDW structure upon heating, a behavior that is rationalized by thermodynamic considerations based on the Clausius-Clapeyron equations and experimental results of the entropy change and the pressure dependence of the transition temperature.

In this study, we now investigated the *c*-axis lattice discontinuity for 1T-TaS_2_ during charge density wave phase transitions by applying atomic force microscopy under ultra-high vacuum (UHV) conditions. By directly monitoring the sample height during the phase transition, our experiments provide a very direct assessment of the *c*-axis discontinuity during the phase transition under previously unexplored conditions, i.e. ultra-high vacuum.

Surprisingly, we found completely opposite effects compared to previous results, i.e., the *c*-axis lattice experiences a contraction during the NC → C transition upon cooling and an expansion during the C → T transition upon heating. Additionally, our results reveal a previously undiscovered sub-structure within the contraction/expansion curves, which can be considered as representative for the initial stages of the phase transition. In the upcoming sections we will now present the experimental results in detail and discuss how they can fit to the common concepts about first order CDW-transitions in 1T-TaS_2_ or if they represent a major paradigm shift requiring consideration of new aspects related to CDW phase transitions.

## Results

**Figure 1:**
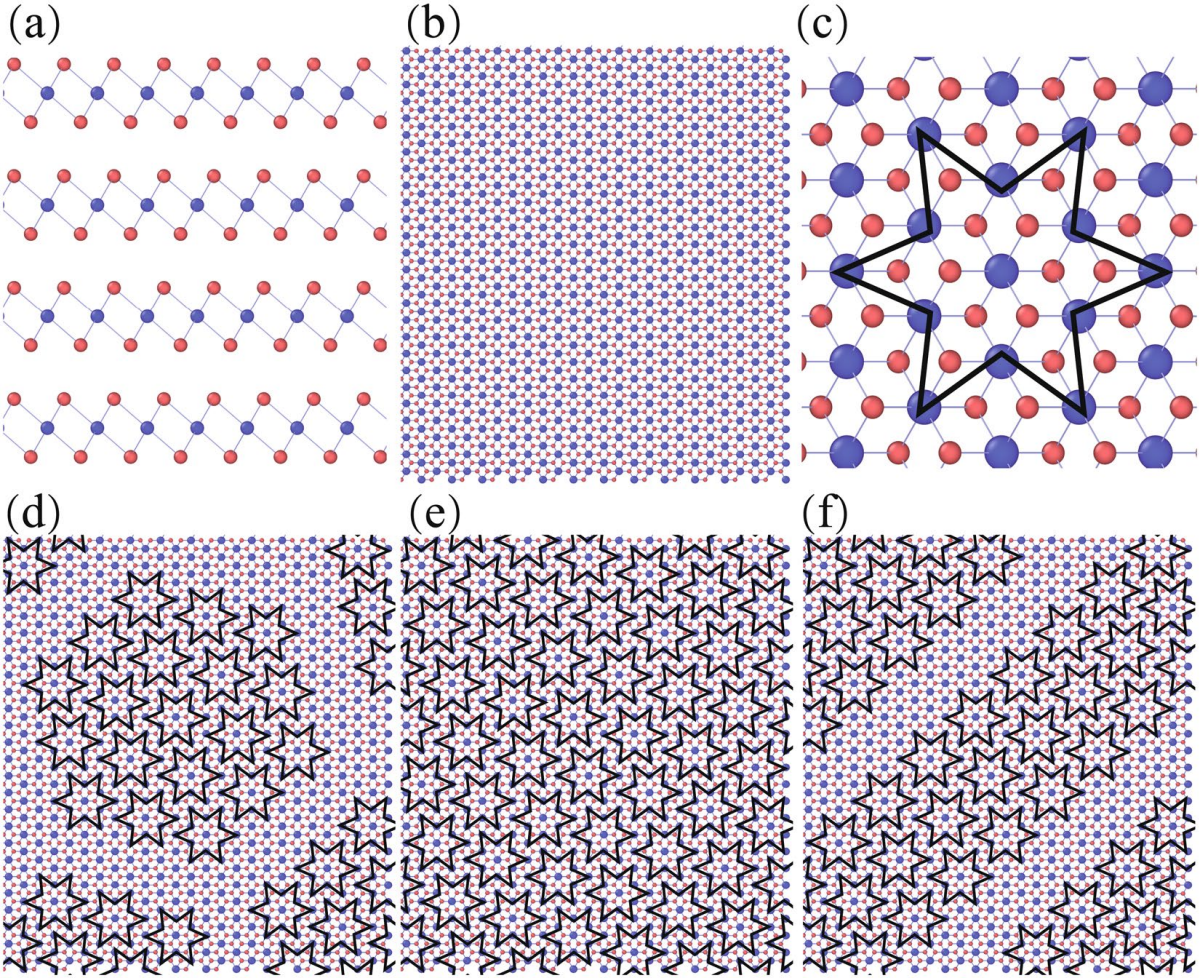
Illustration of the structure of 1T-TaS_2_ at various temperature. (**a**) Side view of 1T-TaS_2_. (**b**) Top view of 1T-TaS_2_. (**c**) Illustration of a ‘star of David’﻿ where 12 Ta atoms within the layer move to the 13th central Ta atom. (**d**) A schematic drawing of NC-CDW phase in which ‘star of David’ clusters formed several separate hexagonal array structures (**e**) Illustration of C-CDW phase, which consists of a uniform distribution of ‘stars of David’. (**f**) Illustration of T-CDW phase consisting of striped triclinic C-CDW domains.

As illustrated in Fig. 1a,b, 1T-TaS_2_ can be described as a regular stack of TaS_2_ tri-layers. Each of these tri-layers has a simple crystalline structure, where a layer of Tantalum (Ta) atoms is sandwiched by two layers of hexagonally packed Sulfur atoms. The Sulfur atoms are located at the corners of an octahedron and connect to the Ta atoms by strong chemical covalent bonds, while the forces between the different tri-layers are based on relatively weak and long range van der Waals interaction^25^.

The principle structure of 1T-TaS_2_ remains stable up to temperatures of about 600 K before an irreversible transition to 2H-TaS_2_ occurs^26^. Cooling down from temperatures below this transition temperature, 1T-TaS_2_ experiences a number of reversible phase transitions: At 543 K the material transforms from a metallic phase to an incommensurate CDW (IC-CDW) where Ta atoms shift slightly from their original positions and thus form an incommensurate CDW phase. Below 347 K, a nearly-commensurate CDW (NC-CDW) phase is formed with partial structures of reorientation being commensurate to the original lattice. More specifically, 12 Ta atoms within the layer move to a 13th central Ta atom and form a structure commonly describes as ‘star of David’^15^ (see Fig. 1b). In the NC-CDW state, clusters of these ‘stars of David’ arrange as hexagonal arrays in commensurate configuration, which are separated from each other by regions without this order (see Fig. 1d). Upon further reduction of the sample temperature below 183 K, a commensurate CDW (C-CDW) phase is formed, where the commensurate hexagonal clusters formed by the 13 atom stars amount to full coverage (Fig. 1e). If the sample temperature is reduced even further, 1T-TaS_2_ can even transform into a Mott insulator or a superconductor depending on the applied external field^1,4,27–31^. If on the other hand, the temperature is increased again, the C-CDW configaration will remain stable until a temperature of about 223 K is reached, at which the system transforms from to a nearly-commensurate triclinic (T-CDW) phase, which is characterized by the commensurate clusters of stars forming distinct stripes across the layers (Fig. 1f). This phase then remains stable, until a temperature of 280 K is reached and a phase transition back to the NC-CDW phase occurs. All these different transitions are accompanied by significant changes in the samples electrical conductivity, as a direct evidence of the close correlation between crystalline structure and electronic band structure^10^.

**Figure 2:**
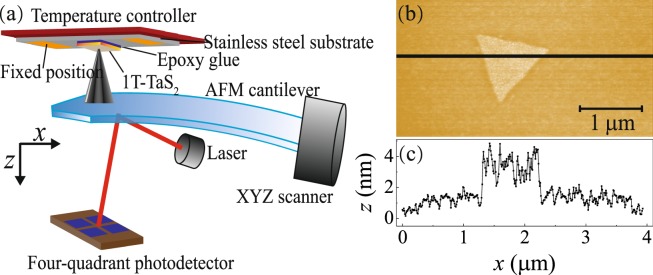
Illustration of the UHV VT-AFM. (**a**) Measurements of the *c*-axis lattice change were made in a UHV VT-AFM in which a flake of 1T-TaS_2_ was glued on a stainless-steel substrate which was mechanically contacted to a temperature controller. Both sides of the stainless-steel substrate were fixed to a stage. (**b**) The topography of an Au particle on 1T-TaS_2_. (**c**) Cross-section through the Au particle shown in (**b**) exhibiting a height of around 3 nm.

To analyze the lattice changes perpendicular to the 1T-TaS_2_ layers during the CDW phase transitions, we have applied contact mode atomic force microscopy which yields an excellent height resolution in the order of 0.1 nm. Details of the experimental set-up are shown in Fig. 2a. All measurements were done under UHV conditions using a commercial Scienta Omicron UHV VT-AFM. To perform our experiments, the 1T-TaS_2_-sample (purchased from HQ graphene) was first glued onto a stainless-steel substrate of 1 mm thickness, which was then mechanically fixed to a sample holder, that was coupled to a flow cryostat cooled by liquid nitrogen. By adjusting both the flow of liquid N_2_ and the power dissipated within an additional resistive heater, we can control the temperature of the sample in a range of appr. 100–300 K.

To verify the correct orientation of the *z*-axis within our AFM, we first deposited Au-nanoparticles onto the sample surface by 600 s of thermal evaporation from a Knudsen-Cell at 1460 K. Figure 2b,c show the topography of such a nanoparticle measured at room temperature by using a standard Si-cantilever with a nominal force constant of 0.2 N/m. As expected, the Au-nanoparticle appears as an elevated structure with a height of about 3 nm. Hence, any measured *z*-displacement in our phase transition experiments can directly be correlated to the *c*-axis lattice change without considering height inversion effects.

In order to obtain a clean sample surface for our phase transition experiments, the 1T-TaS_2_ was freshly cleaved by scotch tape directly before transfer to the UHV chamber. Inside the UHV chamber, the 1T-TaS_2_ was additionally heated to 450 K for 2 h to remove residual adsorption from the surface. To reduce tip-wear during our temperature dependent analysis of the *c*-axis lattice change, all measurements have been performed using a commercial diamond tip cantilever (purchased from Adama Innovations) with a nominal normal force constant of *k* = 0.25 N/m and a typical tip diameter of 10 nm. The basic protocol of our measurements is already outlined in ref.^6^. In principle, the sample is continually scanned while the temperature is simultaneously varied and the *z*-position of the tip is recorded together with the sample temperature. In the case of this work, we used a scanning area of 50 nm × 50 nm and a scanning velocity of *v* = 250 nm/s. At the same time, the rate of temperature change was about 0.9 K/min for both the cooling and the heating processes. Additionally, independent measurements have been performed for different normal forces ranging from 16 nN to 47 nN in order to check for possible influences of local contact pressure exerted by the AFM-tip.

In the upcoming sections, changes of the *c*-lattice-constant *c* will be defined consistently to previous works^26,32,33^ by referring to the changes from the low to high temperature side of the transition, i.e. Δ*c* = *c*_*high*_ − *c*_*low*_ with *c*_*high*_ and *c*_*low*_ representing the lattice constant of the high temperature and low temperature side. Accordingly, changes of the absolute sample height *z* will be defined as Δ*z* = *z*_*high*_ − *z*_*low*_. To calculate the values of Δ*c* from the absolute height changes Δ*z*, we used the absolute thickness of 90.5 ± 19.5 *μ*m of our sample as measured by confocal laser microscopy at room temperature.

**Figure 3:**
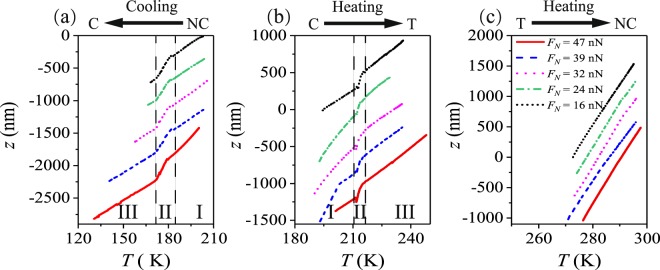
The measured *z*-direction displacement versus temperature at different transition regions with various normal loads from 16 nN to 47 nN. (**a**) *z*-direction displacement during NC → C transition when cooling, (**b**) *z*-direction displacement during C → T transition and (**c**) T → NC transition when heating. Please note, that the AFM does not allow to measure the absolute *z*-height of the sample. Instead, the *z*-value measured for *F*_*N*_ = 16 nN at the beginning of each phase transition has been set to zero, while all other curves have been shifted vertically in order to separate them within the figures.

Figure 3 shows the measured *z*-position of the AFM tip during the three different CDW phase transitions, namely NC → C (a), C → T (b), and T → NC (c). From Fig. 3 it can clearly be seen, that the *z*-displacement experiences a sudden contraction during the NC → C transition when cooling the sample and a sudden expansion during C → T transition upon heating. For the T → NC transition, on the other hand, we cannot attribute any obvious change in z-position to the phase transition. Thus, our focus in the following sections will be directed towards the NC → C and C → T transitions.

In principle, the temperature dependent *z*-displacement curves shown in Fig. 3a,b can each be divided into three regions: (I) a nearly linear dependence of displacement on temperature, which characterizes the predominant effects of thermal expansion or contraction of the sample before the temperature reaches the transition temperature; (II) a sudden increase or decrease of the *z*-displacement, which corresponds to the *c*-axis lattice changes during phase transitions; and (III) a nearly linear dependence of displacement on temperature after the transition is complete and *z*-changes are again dominated by continuous thermal expansion or contraction within the sample or the general experimental setup.

**Figure 4:**
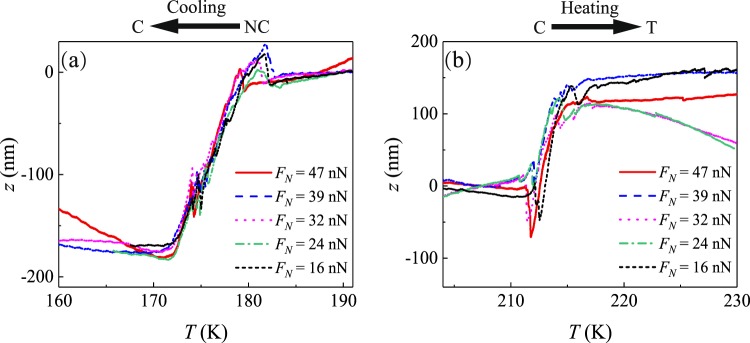
The *z*-direction displacement versus temperature after subtracting the global thermal expansion or contraction within the sample or the general experimental setup at two different transition regions. (**a**) NC → C when cooling, (**b**) C → T when heating.

As a next step, Fig. 4 thus shows the discontinuous *z*-position of the AFM tip in reduced temperature ranges after subtracting the continuous thermal expansion as identified in regions I and III of Fig. 3. As expected for phase transitions affecting the whole bulk of the sample, we do not see any significant influence of contact pressure by the AFM tip. Independent of the normal force, when cooling the 1T-TaS_2_, the NC → C phase transition seems to start at a temperature *T* = 182.5 ± 1.5 K and ends at *T* = 171.0 ± 0.5 K (Fig. 4a). At the beginning of the NC → C transition, the *z*-axis lattice first experienced a sudden expansion with Δ*c*/*c* = Δ*z*/*z* = (−2.0 ± 0.8) · 10^−4^ corresponding to the peaks in Fig. 4a. After that, the *c*-axis lattice continually decreases with temperature. From this decrease, an effective *c*-axis lattice discontinuity for the whole NC → C transition of about Δ*c*/*c* = Δ*z*/*z* = (19.2 ± 4.1) · 10^−4^ can be calculated. Apart from these two main effects, only a small but consistent instability can be observed at *T* ≈ 175 K (see Fig. 4a).

On the other side, when heating the 1T-TaS_2_, the C → T phase transition starts at *T* = 210.9 ± 0.3 K and ends at *T* = 217.2 ± 0.6 K. At the beginning of the C → T transition, the *c*-axis lattice first experiences a sudden contraction with Δ*c*/*c* = (−4.6 ± 2.3) · 10^−4^, similar to the inital peaks observed in Fig. 4a. After this initial contraction, the *c*-axis lattice continuously increases with increasing temperature. From this, the effective *c*-axis lattice discontinuity can be calculated to be Δ*c*/*c* = Δ*z*/*z* = (14.1 ± 3.0) · 10^−4^ for the C → T phase transition. Again, there seems to be an instability at the end of the transition, but this particular effect is not so obvious due to the limited signal to noise ratio. However, to our best knowledge, no information about such additional features, as observed within our phase transition curves (Fig. 4a,b), has previously been available for 1T-TaS_2_.

## Discussion

In order to eliminate the possibility that any discontinuities arise from our experimental set-up, we performed control experiments equivalent to the ones shown in Fig. 3 but using Mica as a sample instead of 1T-TaS_2_. In this case, we did observe an almost linear expansion or contraction without any discontinuities. All in all, these results show, that monitoring the sample height by contact mode atomic force microscopy is a suitable approach to analyze the *c*-axis discontinuities during phase transitions of 1T-TaS_2_. But surprinsingly, our results cannot easily be reconciled with previous macroscopic studies^26,32,33^, which used diffractometry and capacitance dilatometry to characterize the thermal expansion or contraction of 1T-TaS_2_ during phase transitions. All results of the *c*-lattice discontinuity are summarized in Table 1 in comparison with the results of this work (Please note, that Guy *et al*.^33^ just reported the change of the volume, but the conclusion is quantitively consistent with refs^26^ and^32^). A comparison based on the values shown in Table 1 reveals, that the absolute magnitude of the AFM-based values is smaller by a factor 2–3, while at the same time and far more importantly also the algebraic sign of Δ*c*/*c* is different.

**Table 1:** Discontinuities in *c*-axis lattice of 1T-TaS_2_ across NC to C transition when cooling and C to T phase when heating. The sign always refers to the change from low to high temperature side of the transition.

Lattice discontinuity	Δ*c*/*c* [10^−4^]	Δ*c*/*c* [10^−4^]	Δ*c*/*c* [10^−4^]	Δ*V* [10^−2^*cm*^−2^/*mol*]
Experimental method	AFM	Diffractometer	Capacitance dilatometer	Derived from phase-diagram analysis
Experimental conditions	UHV	Helium atmosphere	Helium atmosphere	1 bar - 9.2 kbar
*Cooling*				
NC → C	19.2 ± 4.1	−46	−49 ± 8	−27 ± 2
*Heating*				
C → T	14.1 ± 3.0	−32	−32 ± 4	−8.5 ± 0.8
T → NC	—	—	−7 ± 1	−0.97 ± 0.12
Source	This work	ref.^26^	ref.^32^	ref.^33^

Basically, it was found, that the values reported in refs^26,32,33^ are in good agreement with predictions for a first order phase transition based on the Clausius-Clapeyron-equation *dP*/*dT* = Δ*S*/Δ*V* = *L*/(*T*Δ*V*). Here, the change of volume Δ*V* is directly related to the entropy change Δ*S* and the pressure dependence of the transition temperature *dP*/*dT*. Values of *dP*/*dT* and Δ*S* are available for both NC → C and C → T transitions^32,33^. The reported values of *dP*/*dT* are in the range of −0.08 kbar/K and −0.07 kbar/K for the NC → C transition and between −0.18 kbar/K and −0.12 kbar/K for the C → T transition. Δ*S* is 0.52 ± 0.02 cal/mol/K and 0.33 ± 0.01 cal/mol/K for the NC → C transition and C → T transition respectively. Since *dP*/*dT* is negative and Δ*S* is positive for both transitions, Δ*V* must be negative, which means that the volume of 1T-TaS_2_ is always larger at the low temperature side of both transitions. As we know, Δ*a*/*a*^26,32,33^ is always positive for both transitions (please recall, that Δ*a* and Δ*c* always refer to the change from low to high temperature side of the transition), i.e. heating leads to in-plane expansion and cooling results in in-plane contraction, so Δ*c*/*c* must be negative, as found in the previous publications.

These considerations further emphasize the question, why the AFM-based analysis results in completely opposite phenomena. First of all, an error related to data acquisition or data analysis might be conceivable. However, this aspect was checked carefully and Fig. 2b,c confirm, that the topography of a gold nanoparticle is indeed correctly reproduced without any topography inversion effects. Thus, we can rule out any methodological problem and the effects observed during the phase transition, including also the additional peaks observed at the beginning of each phase transition, must instead rather be related to physical processes intrinsic to our samples. In comparison to the previous studies listed in Table 1, a main difference concerns the environmental conditions, under which the measurements have been performed. On contrary to previous experiment, which have been done under ambient pressure in protective gas environments, we now used UHV conditions. It is well known, that the structural and electronic properties of 1T-TaS_2_ are sensitively depending on pressure^1,34,35^. In this context, it might be speculated, that already comparatively low external pressure can especially influence the rearrangement of the weakly bound TaS_2_ layers, where e.g. more compact stacking (i.e. Δ*c*/*c* > 0) might be expected as a result of the commensurate conditions after the NC → C transition. In case of higher pressures, especially defects might prevent this reordering resulting in *δc*/*c* < 0 as also observed in the initial stages of our phase transitions. In addition to pressure, also the general quality of the lattice structure might be of importance. But while e.g. the 1T-TaS_2_ powder used in ref.^26^ suggests a rather high number of defects, a meaningful comparison to the bulk samples used in our work is not possible.

Although we did not observe any significant load dependence during our measurements, we cannot disregard the fact, that our normal forces ranging from 16–48 nN can still exert a sizeable local pressure in the range of 0.05–0.15 GPa, when considering typical AFM tip radii of appr. 10–20 nm. However, it does not seem feasible that the expansion/contraction effects during the phase transitions are solely related to probing changes of the materials Young’s modulus *E* by the AFM tip. Assuming typical values of *E* in the range of a few GPa, this would require changes of *E* by several 10%, even if a uniform tip induced pressure throughout the whole sample is assumed. Such variations cannot be expected, instead typical changes of the Young’s modulus are limited to appr. 4%^36^.

To conclude, our analysis characterizes the structural transformations, i.e. the *c*-axis lattice discontinuities, of 1T-TaS_2_ during CDW phase transitions. By applying temperature dependent AFM measurements, this could be done for the first time under UHV conditions, where direct and accurate experimental results have previously been unavailable. In the course of our work, a surprising behaviour was revealed: For the NC → C transition, we found that the *c*-axis lattice experiences a sudden expansion at the beginning of phase transition near 183 K and then a quick contraction across NC → C transition during the cooling process. This was reversed during the C → T transition. Here, the *c*-axis lattice first contracts suddenly and then expands quickly. Finally, no obvious lattice discontinuity was observed during the T → NC phase transition. Also, our results do not only show an inversion of the absolute expansion/contraction effects compared to previous publications, but also reveal subtler features of the phase transitions, that were previously unreported. While it can be speculated, that the effects are at least partially related to the low pressure conditions, a detailed theoretical analysis of the phase transition processes might provide new insight into 1T-TaS_2_ first order phase transition processes, that could also prove relevant for the realization of new and improved nanoscale devices based on 1T-TaS_2_.

## Methods

During our experiments we used a commercial 1T-TaS_2_ sample obtained from HQ-Graphene (Groningen, The Netherlands). The sample was mounted on a standard Omicron low temperature sample holder by use of conductive epoxy glue (Massachusetts, USA). All expansion experiments have been performed within the Omicron UHV VT-AFM/STM using PPP-LFMR cantilever obtained from Adama Innovations (Dublin, Ireland). During our measurements an average pressure of about 3 · 10^−10^ mbar was constantly maintained. All measurements have been performed in contact mode operation with normal forces as specified in the main text. During our measurements, the feedback control of the normal force signal was always activated and feedback parameters ensuring fully stable normal forces were chosen. After the AFM measurements have been concluded, we analyzed the sample using a laser microscope (Keyence, Model 9710) to measure the sample height.
